# Adamantinoma of Bone: A Structured Narrative Review of Clinical Outcomes, Recurrence Patterns, and Metastatic Behaviour

**DOI:** 10.3390/reports9030221

**Published:** 2026-07-10

**Authors:** Albara Dabroom, Muhanad Alzahrani, Mohammed Ayed M. Alshammari

**Affiliations:** 1 Department of Orthopedic Surgery, King Abdulaziz Medical City, Ministry of National Guard-Health Affairs, Jeddah 22384, Makkah Province, Saudi Arabia; 2 Ministry of Health, Jeddah 23344, Makkah Province, Saudi Arabia; mohandsaleh50@gmail.com; 3 King Faisal Specialist Hospital and Research Centre, Jeddah 21499, Makkah Province, Saudi Arabia; dr.alshammari91@gmail.com

**Keywords:** adamantinoma, osteofibrous dysplasia, long bone tumour, local recurrence, metastasis, limb salvage, structured narrative review, risk of bias

## Abstract

**Background/Objective:** Adamantinoma is a rare, low-grade malignant primary bone tumour with a predilection for the tibial diaphysis. Despite decades of case series and institutional cohorts, the evidence base remains fragmented, and outcomes are inconsistently reported across studies. To synthesise the best available evidence on clinical outcomes, recurrence patterns, metastatic behaviour, and surgical management of skeletal adamantinoma and to appraise the methodological quality of the contributing literature. **Methods:** A structured narrative review was conducted searching PubMed (294 records) and Web of Science (397 records) from inception to April 2026, yielding approximately 532 unique records after deduplication. Case series of five or more patients with histologically confirmed skeletal adamantinoma were included. A risk-of-bias critique was applied across five domains to each included study. **Results:** In total, 17 studies, representing more than 900 reported patient entries with possible cohort overlap, formed the primary evidence base. Local recurrence rates for classic adamantinoma (AD) range from 15% to 31%, with metastatic rates from 10% to 27%, predominantly to the lung. The osteofibrous dysplasia-like subtype (OFD-AD) showed no metastases in any series that reports this subtype separately but carries a locally aggressive recurrence rate of 22–43%. Wide resection with uncontaminated margins is the most consistently protective surgical variable (hazard ratio 0.164; *p* < 0.001). Late recurrences beyond 15 years are documented in multiple series, supporting prolonged surveillance. An MRI-based model for metastatic risk stratification at diagnosis has been proposed but requires external validation. **Conclusions:** Adamantinoma is more dangerous over a longtime horizon than its low-grade designation implies. Subtype distinction, margin status, and lifelong surveillance are the cornerstones of management. The evidence base carries predominantly moderate to high risk of bias; all conclusions should be interpreted accordingly. A multinational prospective registry remains the most important unmet research need.

## 1. Introduction

Adamantinoma of the long bones is one of the least common primary skeletal malignancies, accounting for an estimated 0.01–0.05% of all primary bone tumours [1,2]. The term “adamantinoma” was coined by Fischer in 1913 because of its histological resemblance to ameloblastoma of the mandible; the two tumour types have no biological or pathogenic relationship [3].

Adamantinomas occur primarily in the diaphysis of the long bones of the lower leg, particularly the tibia. Nearly all reported cases involve the tibia (approximately 82–97%) [1,4]. Ipsilateral fibular co-involvement is noted in approximately 10–15% of cases, and rare examples involving the femur, humerus, ulna, and radius have also been described [1]. Patients usually present in the second to fourth decade of life after a prolonged period of pain and swelling, frequently lasting more than a year before medical attention is sought; the longest reported delay was 50 years, illustrating how slow-growing these tumours tend to be [1]. This indolent growth often results in delayed diagnosis and, in many cases, in initial misdiagnosis as a benign fibro-osseous lesion [5].

Histologically, adamantinoma is a biphasic tumour composed of epithelial cell islands within an osteofibrous stroma. The epithelial component expresses cytokeratins (AE1/AE3, CK5/6) and the squamoid markers p63 and p40, confirming its epithelial lineage [6,7]. Five architectural patterns are recognised: basaloid (most common), tubular, squamous, spindle cell, and cording [1]. A rare dedifferentiated variant carries a more aggressive clinical course [6].

The identification of the osteofibrous dysplasia-like subtype (OFD-AD) by Czerniak and colleagues in 1989 was a pivotal conceptual advance [8]. This entity is characterised by young patient age, intracortical location, predominant osteofibrous dysplasia (OFD)-like stroma, and only scattered cytokeratin-positive cells. It occupies an intermediate position on a biological spectrum between OFD and classic AD [3], and its clinical behaviour, locally aggressive but with no metastases reported in available series, has fundamental implications for treatment strategy and surveillance planning.

Because randomized studies are not possible due to the extreme rarity of the tumour, all data have been obtained from case series, retrospective cohorts, and registry analyses across more than seven decades. This structured narrative review assesses the clinical outcome data, recurrences, metastases, and operative management and evaluates the methodological constraints associated with each contributing study.

## 2. Methods

### 2.1. Study Design

A structured narrative review was conducted to accommodate the heterogeneity of available evidence, spanning institutional case series, retrospective cohorts, registry analyses, clinicopathological studies, and imaging studies, and to enable coherent thematic synthesis with explicit methodological critique.

### 2.2. Literature Search

Searches were performed in PubMed (294 records) and Web of Science (397 records) from database inception to April 2026, yielding approximately 532 unique records after deduplication. The PubMed strategy used (“adamantinoma” [Title/Abstract]) AND (bone OR tibia OR tibial OR skeletal). The Web of Science strategy used TS = (“adamantinoma”) AND TS = (tibia OR tibial OR fibula OR “long bone”) NOT TS = (jaw OR mandib* OR maxill* OR odontogenic OR ameloblastoma). All 532 records underwent title and abstract review. Studies clearly not addressing skeletal adamantinoma or presenting extractable outcome data were excluded at this stage.

### 2.3. Inclusion and Exclusion Criteria

Studies were eligible for inclusion based upon their reporting of original patient-level outcomes in a minimum of five patients diagnosed with histologically confirmed skeletal adamantinoma of any long bone. Studies with fewer than five patients were included only when they provided unique information not available from larger cohorts, such as subtype-specific behaviour, imaging-based risk stratification, or treatment response; these smaller studies were used for qualitative discussion only and did not contribute to the pooled numerical ranges unless their outcomes were explicitly presented separately. The following were excluded from eligibility: single case reports; narrative reviews that did not report on an original cohort; jaw/odontogenic tumour reports; and abstract-only presentations at conferences.

### 2.4. Data Extraction

Two reviewers independently screened titles and abstracts and then full texts against the eligibility criteria, extracted data using a predefined extraction form, and applied the risk-of-bias framework; disagreements were resolved by discussion and consensus. For each included study, the following were extracted: author, year, country, study design, sample size, histological subtype, patient demographics, tumour location, surgical approach, margin status, local recurrence rate, metastatic rate, disease-specific and overall survival, follow-up duration, and reconstruction details, where reported. A summary extraction table is presented (Table 1).

### 2.5. Methodological Quality Assessment

A structured narrative risk-of-bias (RoB) critique was applied to each study across five domains: (i) patient selection and representativeness; (ii) diagnostic consistency and histological verification; (iii) follow-up completeness and duration; (iv) confounding and comparability; and (v) reporting quality and statistical appropriateness. Each domain was rated Low, Moderate, or High against prespecified anchors, and an overall rating (Low, Moderate, Moderate–High, or High) was assigned using a fixed combination rule; the domain definitions, anchors, decision rule, and per-study judgements are provided in the Supplementary Material Tables S1 and S2. Two reviewers applied the criteria independently, with disagreements resolved by discussion. These ratings are integrated within the relevant results sections. A published RoB instrument was not adopted because no validated tool fits the single-arm case series that dominate this literature; the five domains were instead anchored explicitly to ensure reproducibility.

## 3. Results

### 3.1. Overview of Included Studies

The evidence base comprised 17 studies published between 1977 and 2026, together representing more than 900 reported patient entries; because several series originate from overlapping referral centres and two analyses draw on the same national SEER registry, this total should be regarded as patient entries rather than unique individuals. The studies span a range of designs: single- and multi-institution retrospective case series, two population-based SEER analyses, clinicopathological studies, and one MRI-based imaging cohort. Geographically, the cohorts derive predominantly from North America and Europe, with additional series from East Asia, and all were conducted at tertiary academic or referral centres. Most reported on classic adamantinoma; a smaller subset reported the osteofibrous dysplasia-like subtype (OFD-AD) separately, and the largest multicentre series contributed the majority of subtype-stratified data. Sample sizes ranged from fewer than ten to several hundred patients, and reported follow-up ranged from under three years to several decades, a heterogeneity that directly informs the risk-of-bias assessment.

### 3.2. Epidemiology, Demographics, and Tumour Characteristics

Adamantinoma is estimated to affect fewer than one person per million per year. The two available SEER analyses identified 92 and 74 patients, respectively, across periods spanning 1973 to 2016 and beyond [17,19], reporting a mean or median age at diagnosis of approximately 20 to 31 years. The largest surgical cohort recorded 53% female across 318 cases [4]. Both SEER analyses rely on ICD-O-3 coding without histological verification, and OFD-AD is not separately captured; their estimates should be interpreted as directional rather than definitive.

The age distribution differs markedly by subtype: mean age at diagnosis was 17 years for OFD-AD versus 32 years for classic AD in the Schutgens et al. multicentre analysis [4], consistent with the paediatric-predominant OFD-AD age distribution reported by Gleason et al. [21]. Tibial involvement is present in 82% to 97% of cases, with the anterolateral diaphysis predominating [1,4]. Multifocality occurs in approximately 24% of cases [4]. Pathological fracture at presentation was recorded in 10% to 21% across series [1,2,4] and independently predicts local recurrence (HR 1.97; *p* = 0.028) [4].

### 3.3. Histological Classification and the OFD–AD Spectrum

Adamantinoma has been extensively studied in terms of its histologic variation by Keeney et al. to determine whether specific histologic patterns would be predictive of an individual patient’s outcome. Only one histologic factor correlated with an increased recurrence rate (i.e., lack of squamous differentiation) in this study of 85 patients [1]. Epithelial lineage can be confirmed via cytokeratin staining, which is commonly used for diagnostic purposes. Studies have shown broad cytokeratin staining (keratins 14 and 19) in all specimens examined (Hazelbag et al.) [22], as well as AE1/AE3 staining in 95% of 28 patients evaluated in another study (Jayan et al.) [7].

**Figure 1:**
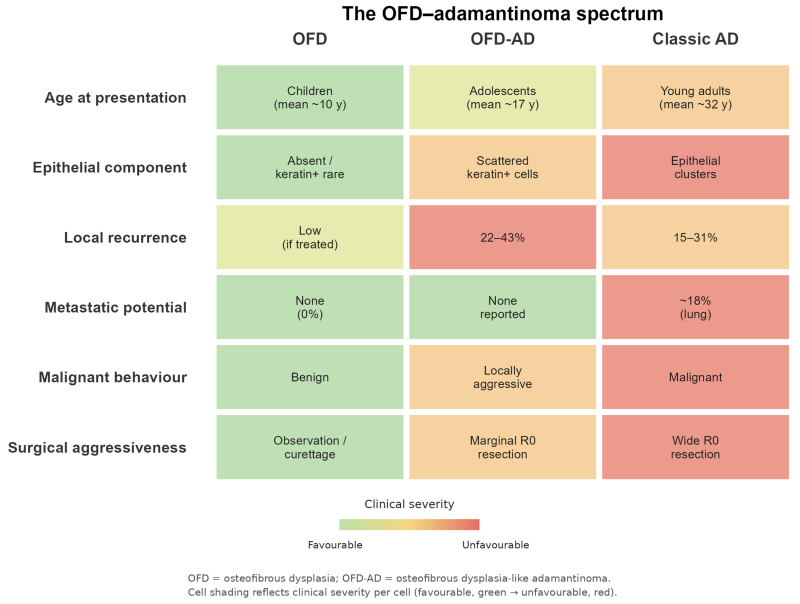
The OFD–adamantinoma biological spectrum. Local recurrence: OFD-AD 22–43%; classic AD 15–31% [1,2,4,11,15]. No metastases have been reported in OFD or OFD-AD in available subtype-stratified series; metastasis is confined to classic AD (~18%, predominantly lung) [4]. Cell shading reflects clinical severity per cell (favourable, green; unfavourable, red). OFD is a benign, cytokeratin-negative lesion of childhood with a moderate local recurrence rate and no metastases reported in available series; conservative management or observation is appropriate. OFD-AD is locally aggressive, with a high local recurrence rate (22–43%) but no metastases reported across published series; marginal R0 resection is recommended. Classic AD requires wide e nbloc resection given its metastatic potential (10–27%) and significant disease-related mortality at 30 years [4,11,25]. Metastatic behaviour: no metastases have been reported in OFD or OFD-AD in any subtype-stratified series [4,16,25,26]. Abbreviations: AD = classic adamantinoma; OFD =osteofibrous dysplasia; OFD-AD = osteofibrous dysplasia-like adamantinoma.

Czerniak et al. established the first formal definition of the OFD-like variant of AD in their 1989 publication (Figure 1) [8]. They identified several key features of this subgroup based on their studies. These characteristics include being less than 20 years old when diagnosed, having an intracortical site of origin, showing OFD-like patterns of stroma with scattered cytokeratin positive cells, and distinguishing it from OFD, which does not contain cytokeratin-positive cells [23], and classic AD. The existence of these distinctions is also acknowledged in the most recent WHO classification system [6], as well as supported by molecular data from Ali et al., who showed that they could separate the transcriptomic profiles of OFD-AD from those of classic AD [24].

Evidence from the largest available dataset documents only one case of OFD-AD progressing to classic AD in 318 patients over 29 years of untreated observation [4]. Neither Scholfield et al. [12], Ramkumar et al. [25], Chen et al. [26], nor Jayan et al. [7] observed progression in their respective series. Diagnostic misclassification remains clinically significant: Jayan et al. revised 20.7% of diagnoses on expert review [7], and Szendroi et al. found that 6 of 11 patients had been misdiagnosed at the referring institution [13]. Springfield et al. similarly reported diagnostic revision in 19 of 32 cases reviewed at a single institution [10]. Expert bone pathology review with a full immunohistochemical panel is therefore essential before definitive treatment planning.

A methodological note applies to series predating the formal recognition of the OFD-AD subtype by Czerniak et al. in 1989, including the foundational Keeney [1] series, which did not separate OFD-AD from classic AD. Their aggregate outcome rates therefore incorporate a proportion of less aggressive lesions and cannot be directly compared to subtype-stratified data from later series such as Hazelbag et al. [11] and Schutgens et al. [4].

### 3.4. Surgical Management and Reconstruction

The shift from amputation-first to limb-salvage surgery was consolidated by Qureshi et al. international multicentre study reporting 84% limb salvage across 23 centres [2], supported by the comprehensive surgical review by El Beaino et al. [27]. Wide en bloc resection with uninvolved margins, including the periosteum, is now the widely accepted standard of care for classic AD [4,6].

Surgical margin status is the dominant predictor of local recurrence. Qureshi et al. demonstrated that wide margins significantly reduced recurrence (*p* < 0.00001) [2]. Keeney et al. reported zero recurrence or metastasis among 12 patients receiving strictly defined wide excision [1]. Schutgens et al. confirmed contaminated margins as the dominant risk factor (HR 0.164 for uncontaminated margins; *p* < 0.001), with periosteal involvement in 38.8% of cases [4].

Curettage is contraindicated for classic AD: all curettage-treated patients recurred in both Szendroi et al. [13] and Puchner et al. [14]. On univariable analysis, intralesional resection carried an HR of 4.18 for local recurrence in the Schutgens analysis [4]. For OFD-AD, marginal R0 resection is acceptable with the patient counselled regarding a 22% to 43% LR rate [4,16]. Primary amputation confers no survival advantage; Houdek et al. found associations with increased metastasis (HR 3.63; *p* = 0.04) and death (HR 6.10; *p* = 0.007), almost certainly reflecting selection bias [15].

Reconstruction complications are a major source of morbidity. Qureshi et al. reported a 48% complication rate, with nonunion (24%) and graft fracture (23%) being the most frequent [2]. Houdek et al. documented a 37% complication rate and 39% reoperation rate [15]. No comparative evidence guides reconstruction technique selection; vascularised fibular grafting is advocated where feasible [15]. Functional outcome data are absent from all included series, a significant gap in the evidence base.

### 3.5. Local Recurrence: Rates, Risk Factors, and Temporal Patterns

Local recurrence rates for classic AD range from 15% to 31% across surgical series [1,2,4,15,16]. The 24% rate in the Schutgens et al. multicentre analysis represents the most statistically reliable contemporary estimate [4]. Weiss and Dorfman’s early nine-case series reported an 11% rate, though this predates limb-salvage era treatment standards [9]. OFD-AD exhibits paradoxically higher local recurrence (22 %to 44%), attributable to more conservative initial surgical approaches [4,25]. Hazelbag et al. 32-patient series demonstrated a 43% LR rate for OFD-AD treated predominantly by intralesional surgery [11]. Zumarraga et al. consecutive tibial series of seven cases reported zero local recurrence, likely reflecting the small sample and high rate of clear margins achieved [28].

On multivariable analysis, the only study to model independent predictors of local recurrence (LR) was Schutgens et al., which identified margin contamination at resection (uncontaminated margins: hazard ratio 0.164 versus contaminated margins), female sex as protective (hazard ratio 0.54 versus male), and pathological fracture (hazard ratio 1.97) as independently associated with LR [4]. Additional associations have been reported on univariable analysis only: intralesional resection (hazard ratio 4.18; Schutgens et al. [4]); male sex (hazard ratio 5.92), and surgery at an age older than 20 years (hazard ratio 7.68) in Houdek et al. [15]; perioperative tumour rupture/spill was associated with a higher cumulative incidence of LR on unadjusted analysis in Schutgens et al. but was not entered into the multivariable model [4]. Notably, patient sex has been identified as a predictor of local recurrence in several series, a finding that is not readily explained by known biological differences.

The time course of local recurrence is also unusual. Every study with sufficiently long follow-up has documented recurrences occurring more than 10 years after surgery. Recurrences developing beyond 19 years were reported by Keeney et al. [1]; Houdek et al. noted three recurrences beyond 15 years [15]; Puchner et al. recorded recurrences at 16 and 20 years [14]. These observations indicate that patients require lifelong follow-up, as no evidence-based safe discharge interval can be established from the existing data. Reported local recurrence rates are therefore highly dependent on follow-up duration: the mean follow-up of 35 months reported by Shimizu et al. [18] is inadequate to capture late recurrences in adamantinoma and will systematically underestimate the true local recurrence rate.

### 3.6. Metastatic Behaviour, Distant Disease, and Survival

Metastatic disease is reported almost exclusively in classic AD and is the principal cause of disease-related death. Metastatic rates range from 10% [2] to 27% [15] in modern surgical cohorts, with 18% in the Schutgens et al. multicentre analysis [4] and 32% in the Hazelbag et al. series treated in an earlier era [11]. Lung is the primary metastatic site; however, Houdek et al. reported abdominal or pelvic involvement in 42% of metastatic patients [15], and a vertebral metastasis mimicking a benign Schmorl’s hernia has been documented radiologically [20]. Prior MRI studies including Van der Woude et al. described hig-hsignal intensity on T1 and T2 sequences and intense enhancement but found no prognostic correlation with imaging features [29]. Because metastatic spread is not confined to the lung, chest CT alone may be insufficient for surveillance in selected high-risk patients, in whom staging beyond the chest should be considered; the optimal imaging modality, extent, and frequency of surveillance remain undefined.

No metastases have been reported in OFD-AD in any series that reports this subtype separately, including 128 OFD-AD cases in Schutgens et al. [4]. This is the most consistent subtype-specific finding reported across the literature. The LR-to-metastasis pathway is well established: Keeney et al. demonstrated 9 of 13 metastatic patients had prior LR, with a markedly higher lung-metastasis rate in patients with prior recurrence than in those without (4.2 versus 0.87 per 100 patient-years; *p* = 0.005) [1], and Schutgens et al. confirmed 20 of 24 metastatic patients had prior LR [4]. Achieving local control at index surgery is therefore the primary means of preventing distant failure.

**Figure 2:**
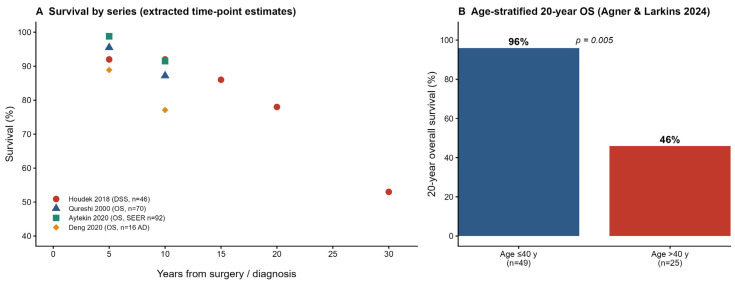
Survival outcomes in adamantinoma of bone. Panel (**A**): The Houdek et al. 2018 series (solid red, *n* = 46) is the only dataset providing disease-specific survival (DSS) data to 30 years, demonstrating a decline to 53% at 30 years, all attributable to tumour recurrence [15]. The Deng et al. 2020 series [16] and Qureshi et al. 2000 series [2] provide shorter-term estimates. The Aytekin et al. 2020 SEER analysis [17] reports favourable overall survival, likely overestimating prognosis due to population-level case mix and absence of histological verification. The 50% reference line (dotted) is provided for clinical context. Panel (**B**): Age-stratified 20-year overall survival from the Agner and Larkins 2024 SEER analysis (*n* = 74) [19]. Patients aged 40 years or below (*n* = 49) had a 20-year OS of 96% versus 46% in those aged above 40 (*n* = 25; *p* = 0.005). All values shown are timepoint estimates extracted from the published reports rather than reconstructed Kaplan–Meier curves; because the series differ in endpoint (OS versus DSS), sample size, and follow-up duration, they are not directly comparable and are presented only as an evidence summary. Abbreviations: DSS = disease-specific survival; OS = overall survival; SEER = Surveillance, Epidemiology, and End Results Program.

Short- and medium-term survival is generally favourable: As shown in Figure 2, SEER analyses report 5-year OS of 98.8% and 10-year OS of 91.5% [17]. However, Houdek et al. 30-year Kaplan–Meier data demonstrate disease-specific survival declining from 92% at 10 years to 78% at 20 years and 53% at 30 years, with all deaths attributable to tumour recurrence [15]. Agner and Larkins documented that patients over 40 years at diagnosis had a 20-year OS of only 46% versus 96% in those aged 40 or below (*p* = 0.005) [19]. These long-term data confirm that adamantinoma’s low-grade designation can create a false impression of clinical safety.

## 4. Discussion

This review’s most consistent finding is that adamantinoma is a more dangerous disease over a longtime horizon than its low-grade malignancy designation implies and that the OFD-AD/classic AD distinction is now sufficiently established to drive differentiated management.

The absence of any reported metastasis in OFD-AD across 128 cases in Schutgens et al. [4] and confirmed across every subsequent series [12] justifies treating OFD-AD and classic AD as effectively separate diseases for staging, surgical planning, and surveillance intensity. The current WHO classification reflects this distinction [6], and ongoing advocacy for reclassification of OFD-AD from a malignant to a locally aggressive category is consistent with the available evidence.

Surgical margin status at index surgery is the dominant modifiable determinant of outcome. The convergent message from Keeney et al. [1], Qureshi et al. [2], Schutgens et al. [4], and Hazelbag et al. [11] is unambiguous: contaminated margins dramatically increase LR risk, and prior LR is the strongest driver of subsequent metastatic disease. Referral to a specialised bone tumour centre before any surgical intervention, including biopsy, is therefore strongly supported by this evidence base.

The longtime horizon of this disease demands a surveillance philosophy different from most bone sarcomas. The combination of documented recurrences beyond 20 years, a 30-year DSS of 53% [15], and the absence of any safe discharge interval must inform patient counselling from the time of diagnosis. The Houdek et al. surveillance protocol is the most detailed published approach and represents a reasonable starting point pending prospective evaluation. It must be emphasised, however, that the optimal surveillance schedule, imaging modality, and duration remain undefined and that no surveillance protocol has been prospectively validated in this disease [15].

The Simonetti et al. MRI model [20] addresses the critical absence of a histological grading system by proposing baseline imaging features as metastatic risk stratifiers. A two-feature model achieved 100% sensitivity and 93.75% specificity in 22 patients. However, the confidence intervals are very wide (vascular invasion OR 121; 95% CI 4.28 to 3424), the study is from a single centre, and 5 of 22 patients lacked contrast-enhanced MRI sequences. External multicentre validation is essential before clinical adoption. In the systemic setting, a single metastatic case responding to sunitinib suggests targeted therapy may merit prospective evaluation [30].

### Limitations

This review has several inherent limitations. As a structured narrative rather than systematic review, it is subject to selection bias and does not provide a formal PRISMA flow diagram or GRADE evidence assessment. Included studies are predominantly from tertiary academic centres in high-income countries. The evidence base carries predominantly moderate to high risk of bias: the best-available study [4] is retrospective, spans 30 years, lacks central histological review for the majority, and has 19% missing local recurrence data. Functional outcome data are absent from all series. The search was restricted to PubMed and Web of Science; Embase, Scopus, and the Cochrane Library were not searched, and some older or non-indexed orthopaedic oncology series may therefore have been missed. In addition, several included cohorts derive from overlapping referral centres, multicentre collaborations, or registry datasets, so the figure of more than 900 patients may include a degree of patient overlap that could not be formally quantified. A multinational prospective registry with standardised definitions for subtype, margin status, recurrence, and functional outcomes is the most pressing research need in this field.

## 5. Conclusions

Adamantinoma of bone is a rare but genuinely malignant tumour whose low-grade designation can create a false sense of clinical safety. Classic AD carries a local recurrence rate of 15% to 31%, a metastatic rate of 10% to 27%, and a 30-year disease-specific survival of approximately 53%. OFD-AD is biologically distinct, locally aggressive but with no metastases reported to date, and can be managed with greater surgical conservatism. Wide resection with clear margins remains the key modifiable treatment factor for classic adamantinoma. Because late recurrence and disease-related mortality may occur decades after treatment, prolonged surveillance is justified, although the optimal schedule and imaging strategy remain undefined. Future progress will require prospective multicentre registry data with standardised reporting of subtype, margin status, recurrence, metastasis, survival, and functional outcomes.

## Figures and Tables

**Table 1:** Summary data extraction table for included studies.

Author [Ref]	N	Design	Subtype	Mean Age (y)	Follow-Up	LR (%)	Metastasis (%)	DSS/OS	RoB
Huvos & Marcove, 1975 [5]	14	Case series	Mixed	13–67 (range)	Mean 12 y	71	14	14% fatal	High
Weiss & Dorfman, 1977 [9]	9	Case series	Mixed	38	Variable	11	22	11% fatal	High
Keeney et al., 1989 [1]	85	Retrospective cohort	Mixed (pre-subtype)	25.9	Mean 9 y	31	15% lung (*n* = 13); 7% LN (*n* = 6)	13% fatal	Moderate
Czerniak et al., 1989 [8]	25	Clinicopathological	Classic + OFD-like	Classic 40; OFD-like 11	NR	NR	NR	NR	N/A
Springfield et al., 1994 [10]	32	Retrospective cohort	Spectrum	NR	NR	AD 67%	NR	NR	Mod–High
Hazelbag et al., 1994 [11]	32	Retrospective cohort	Classic AD + OFD-AD	Classic 28.7; OFD-AD 22	Mean 10 y	Classic 24%; OFD-AD 43%	Classic 32%	Classic 28% fatal	Moderate
Qureshi et al., 2000 [2]	70	Multicentre retrospective	Classic AD	31	Median 7 y	18.6 at 10 y	10	87.2% at 10 y	Moderate
Scholfield et al., 2017 [12]	31	Retrospective cohort	AD + OFD-AD + OFD	Classic AD 38; OFD-AD 13	Varied	AD 29%; OFD-AD 30%	AD 43%	AD 33% fatal	Mod–High
Szendroi et al., 2009 [13]	11	Retrospective cohort	Classic AD	29.3	Mean 12.7 y	36	9	9% fatal	Mod–High
Puchner et al., 2016 [14]	15	Retrospective cohort	AD + OFD	28 (AD)	Mean 16 y	AD 40%; OFD 40%	AD 20%	AD 10% fatal	Mod–High
Houdek et al. 2018 [15]	46	Retrospective cohort	Classic AD	24	Mean 16 y	15	27	30 y DSS 53%	Moderate
Schutgens et al., 2020 [4]	318	Multicentre retrospective	AD + OFD-AD	AD 32; OFD-AD 17	Median 83 mo	AD 24%; OFD-AD 22%	AD 18%; OFD-AD 0%	AD 9% fatal	Moderate
Deng et al., 2020 [16]	23	Retrospective cohort	AD + OFD-AD	NR	OFD-AD 66 mo	AD 19%; OFD-AD 14%	AD 12.5%; OFD-AD 0%	AD 10 y 77.1%	Mod–High
Aytekin et al., 2020 [17]	92	SEER registry	Classic AD	30.8	Mean 138 mo	NR	NR	5 y 98.8%; 10 y 91.5%	Moderate
Shimizu et al., 2024 [18]	38	National registry	Classic AD	37	Mean 35 mo	NR	NR	37/38 NED	Mod–High
Agner & Larkins, 2024 [19]	74	SEER registry	Classic AD	20–24 (median)	20 y endpoint	NR	NR	20 y OS: ≤40 y 96%; >40 y 46%	Moderate
Simonetti et al., 2025 [20]	22	Imaging cohort	Classic AD	27 (median)	Median 62 mo	NR	27.3	18.2% fatal	Mod–High

AD = classic adamantinoma; OFD-AD = osteofibrous dysplasia-like adamantinoma; OFD = osteofibrous dysplasia; LR = local recurrence; DSS = disease-specific survival; OS = overall survival; NR = not reported; NED = no evidence of disease; LN = lymph node; RoB = risk of bias; Mod = moderate; N/A = Not Applicable.

## Data Availability

All data were obtained from previously published articles. Extracted data are available from the corresponding author upon reasonable request.

## Supplementary Materials

The following supporting information can be downloaded at: https://www.mdpi.com/article/10.3390/reports9030221/s1, Table S1: Domain definitions and rating anchors; Table S2: Risk-of-bias judgements by study.
