# Supplementary material for: Adamantinoma of Bone: A Structured Narrative Review of Clinical Outcomes, Recurrence Patterns, and Metastatic Behaviour
Source: Reports (MDPI). 2026 Jul 10;9(3):221. doi: 10.3390/reports9030221 (PMC13398085; doi:10.3390/reports9030221)
Supplement: Supplementary file 1 [file reports-09-00221-s001.zip › reports-4297889-supplementary.pdf]

Supplementary Material — Risk-of-Bias Assessment

Adamantinoma of Bone: A Structured Narrative Review of Clinical Outcomes, Recurrence Patterns, and Metastatic Behaviour

Methods (for inclusion in Section 2)

Because the included studies were single-arm case series, retrospective cohorts, and registry analyses for which no single validated risk-of-bias instrument is well suited, each study was appraised across five pre-specified domains: (D1) patient selection and representativeness; (D2) diagnostic verification; (D3) completeness and duration of follow-up; (D4) control of confounding and comparability; and (D5) reporting quality and statistical appropriateness. Each domain was rated Low, Moderate, or High risk against the explicit anchors set out below, so that ratings are reproducible rather than impressionistic. An overall judgement was then assigned using a fixed decision rule: Low overall if no domain was rated High and at least three domains were Low; Moderate if no domain was High; Moderate-High if exactly one domain was High; and High if two or more domains were High. Two reviewers applied the criteria independently and disagreements were resolved by discussion. The assessment was performed against the primary publications.

Table S1 Domain definitions and rating anchors

Domain	Low risk	Moderate risk	High risk
**D1 — Patient selection / representativeness**	Consecutive or population/registry-based series with explicit, reproducible inclusion criteria.	Consecutive single-centre series (referral selection toward complex cases) or minor selection concerns.	Selected/convenience sample, or selection process not described.
**D2 — Diagnostic verification**	All cases histologically confirmed with immunohistochemistry and/or central/expert pathology review.	Histologically confirmed but without central re-review, or review on a subset only.	Diagnosis not uniformly confirmed, or subtype (AD vs OFD-AD) not distinguished where relevant.
**D3 — Follow-up completeness / duration**	Mean/median follow-up >~10 years with little loss to follow-up.	Follow-up ~5–10 years, or partial loss to follow-up.	Follow-up <~3 years, substantial loss, or follow-up not reported.
**D4 — Confounding / comparability**	Multivariable adjustment for key prognostic factors.	Univariable comparisons only.	Outcomes reported without any risk-factor analysis, or estimates uninterpretable (e.g. extreme-width CIs).
**D5 — Reporting quality / statistics**	Outcomes, denominators, and effect estimates with precision all completely reported.	Some denominators, time-points, or precision missing.	Selective or incomplete reporting of key outcomes.

Overall rule: Low = no High and ≥ 3 Low; Moderate = no High; Moderate-High = exactly one High; High = ≥ 2 High.

Table S2 Risk-of-bias judgements by study

Study (ref)	D1 Selection	D2 Diagnosis	D3 Follow-up	D4 Confounding	D5 Reporting	Overall
Weiss & Dorfman 1977	High	Moderate	Moderate	High	Moderate	High
Czerniak 1989	High	Low	High	High	High	High
Keeney 1989	Moderate	Moderate-High	Moderate	Moderate	Low	Moderate
Hazelbag 1994	Moderate	Low	Moderate	Moderate	Low	Moderate
Qureshi 2000	Low	Moderate	Moderate	Moderate	Low	Moderate
Szendroi 2009	Moderate	Moderate	Low	High	Moderate	Moderate-High
Puchner 2016	Moderate	Low	Low	High	Moderate	Moderate-High
Scholfield 2017	Moderate	Low	Low	Moderate	Low	Moderate
Houdek 2018	Moderate	Moderate	Low	Moderate	Low	Moderate
Aytekin 2020	Low	High	Low	High	High	High
Deng 2020	Moderate	Low	Moderate	Moderate	Moderate	Moderate
Schutgens 2020	Low	Moderate	Moderate	Low	Low	Moderate
Agner 2024	Low	Moderate	Low	High	High	High
Shimizu 2024	Low	Moderate	High	High	Moderate	High
Simonetti 2025	Moderate	Low	Moderate	High	Moderate-High	Moderate-High

Ramkumar 2026	Moderate	Moderate	Moderate	Moderate	Moderate	Moderate
Jayan 2026	Moderate	Low	High	High	Moderate	High

Studies are ordered chronologically. D1, patient selection; D2, diagnostic verification; D3, follow-up; D4, confounding/comparability; D5, reporting quality.

Two studies cited in the review (Chen 2025 ; Ali 2019 []) contribute molecular/descriptive data without clinical outcome series and were not rated for outcome-level bias.
